# Downregulation of LRP/LR with siRNA inhibits several cancer hallmarks in lung cancer cells

**DOI:** 10.1002/2211-5463.13544

**Published:** 2023-01-13

**Authors:** Monique J. Bignoux, Tyrone C. Otgaar, Martin Bernert, Stefan F. T. Weiss, Eloise Ferreira

**Affiliations:** ^1^ School of Molecular and Cell Biology University of the Witwatersrand Johannesburg South Africa

**Keywords:** apoptosis, LRP‐LR, Lung cancer, RPSA, telomerase

## Abstract

The incidence and mortality rates of cancer are growing rapidly worldwide, with lung cancer being the most commonly occurring cancer in males. Human carcinomas circumvent the inhibitory pathways induced by DNA damage and senescence through the upregulation of telomerase activity. The 37 kDa/67 kDa laminin receptor (LRP/LR) is a cell surface receptor which plays a role in several cancer hallmarks, including metastasis, angiogenesis, cell viability maintenance, apoptotic evasion, and mediating telomerase activity. We have previously shown that the knockdown of LRP/LR with an LRP‐specific siRNA significantly impedes adhesion and invasion, induces apoptosis, and inhibits telomerase activity in various cancer cell lines *in vitro*. Here, we investigated the effect of downregulating LRP/LR with LRP‐specific siRNA in A549 lung cancer cells. Downregulation of LRP/LR resulted in a significant decrease in cell viability, migration potential, and telomerase activity, as well as a significant increase in apoptosis. Proteomic analysis further suggested the re‐establishment of immune control over the lung cancer cells, a previously unidentified facet of LRP downregulation in cancer. Altogether, we suggest that targeting LRP/LR for downregulation may have therapeutic potential for inhibiting several cancer hallmarks.

## Graphical abstract

LRP/LR is overexpressed in many cancer cell types and is associated with various cancer‐promoting processes. In this study, we downregulated LRP/LR in lung cancer cells and showed that this strategy has a four‐fold function in impeding key cancer hallmarks in the lung cancer cells, which therefore has the potential for improving the prognosis of those suffering from lung cancer.

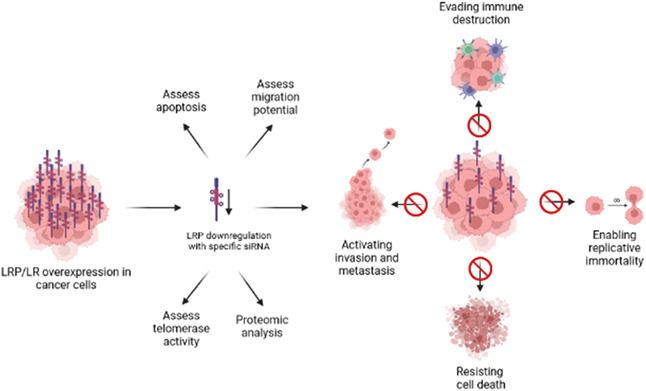

**Abbreviations**
LRP/LR, RPSA, LAMR1: 37 kDa/67 kDa Laminin receptor precursor/laminin receptor, Ribosomal protein SA
NSLC: non‐small‐cell lung cancer
NTC: non‐targeting control
PCA: protocatechuic acid
TRAP: telomeric repeat amplification protocol

Lung cancer is the most common cancer, accounting for 11.6% of reported cases and causing a staggering 18.4% of total cancer deaths in 2018, making lung cancer the leading cause of cancer deaths globally [1]. While there is a 50% 5‐year post‐operative patient survival rate when detected in the earlier stages (stage I/II) of non‐small‐cell lung cancer (NSCLC), more than 50% of patients are only diagnosed at later stages (stage IV) and this is coupled with an appalling 5‐year survival rate of only 2% [2]. Therefore, these statistics highlight the need for effective mechanism‐based targeted therapies for the treatment of lung cancer.

Without a doubt, the proposed hallmarks of cancer, devised and originally described by Hanahan and Weinberg [3, 4], have provided a solid foundation on which to base a logical understanding of neoplasticism. Although these distinct hallmarks have been categorised as such, it is vital to understand that while there are some distinct features, for the most part, the mentioned hallmarks do not act independently. There is co‐dependence and complementarity which exists amongst the hallmarks [4, 5] and as such, it is vital that any further progression in understanding the basis to neoplastic change in its entirety, as well as on a cell‐type‐specific level, needs to take this into account. Therefore, the hallmarks need to be targeted as a whole, rather than individually, for the prospective treatment of cancer.

Due to the many roles it plays in the cell, the 37 kDa/67 kDa laminin receptor (LRP/LR) has become an increasingly popular target in the search for cancer therapeutics due to its characteristic overexpression in many cancer types. In particular, the overexpression of the receptor has been shown to aid in numerous tumourigenic processes, including apoptotic evasion and cell viability maintenance, enhancement of angiogenic processes, as well as enhancing the adhesive and invasive potential of cancer cells [6, 7, 8]. Furthermore, LRP/LR does not only serve as a receptor on the cell surface but also plays a role in intracellular processes through its nuclear and cytosolic localisation. These additional roles include regulation of the cell cycle, ribosomal anchorage to microtubules, protein synthesis, and pre‐rRNA processing [8], as well as in regulating telomerase activity [9, 10, 11]. LRP/LR plays a role in mediating telomerase activity in breast cancer cells and knockdown of LRP/LR with LRP‐specific siRNA significantly reduces telomerase activity [9]. In addition, it was shown that overexpressing LRP/LR enhances telomerase activity [10, 12, 13]. Telomerase is a ribonucleoprotein with reverse transcriptase activity and is principally responsible for elongating telomeres, which are tandemly repeated TTAGGG repeats found capping the ends of chromosomes to aid in preventing chromosomal degradation and the loss of coding DNA [14]. Since human carcinomas circumvent senescence and inhibitory pathways of proliferation induced by DNA damage, through the upregulation of telomerase activity [15], downregulation of LRP with subsequent suppression of telomerase activity is therefore another way in which LRP/LR can be targeted as a potential therapeutic intervention for cancer. The overexpression of LRP/LR in cancer cells allows these cells to exploit the various functions of LRP/LR to aid in the tumourigenic process and LRP/LR thus provides a putative target for the inhibition of multiple cancer hallmarks.

Various studies have observed that targeting LRP/LR for downregulation with LRP‐specific siRNA successfully inhibits numerous tumourigenic processes, as mentioned above, in several different cancer cell types including breast (MDA‐MB231 and MCF‐7) [16], cervical (HeLa) [17], neuroblastoma (IMR‐32), pancreatic (AsPC‐1) [18], oesophageal (WHC01) [16], melanoma (A375 and A375M) [19], and colorectal (SW‐480 and DLD‐1) [20].

The current study therefore serves to extend these results and look further into the effects of downregulating LRP/LR on cancer hallmarks in A549 lung cancer cells, as we have previously shown they exhibit increased LRP/LR levels compared with non‐tumourigenic cells [17, 21]. This includes assessing the effect of LRP downregulation on cell viability and morphology, apoptosis, migration potential, and telomerase activity. Moreover, proteomic analysis was performed to provide insight into the pathways involved after LRP downregulation.

## Materials and methods

### Tissue culture

A549 cells [American Type Culture Collection (ATCC, Manassas, Virginia, USA)] were cultured in Dulbecco's modified Eagle's medium (DMEM) with high glucose (4.5 g·L^−1^) containing 4 mm l‐Glutamine (Sigma‐Aldrich, St. Louis, Missouri, USA), supplemented with 10% fetal bovine serum (FBS; Sigma Aldrich) and 1% penicillin/streptomycin (Sigma Aldrich). Cells were maintained under physiological conditions including 5% CO_2_ at 37 °C in a humidified atmosphere.

### Cell transfection

The A549 cells were transfected in 24‐well plates at 60% confluency with 0.5 μg per well of the LRP‐specific siRNA (MISSION^®^ esiRNA‐RPSA, Sigma Aldrich; EHU109791), as well as with a non‐targeting control (NTC) siRNA (MISSION^®^ siRNA Universal Negative Control #1, Sigma Aldrich; SIC001) for 24–72 h. Mock transfections, containing the transfection reagent in the serum‐free medium only (and no siRNA), were also performed as above to ensure the transfection reagent was not causing any off‐target affects. Transfections were performed using the X‐tremeGENE™ siRNA Transfection Reagent (Roche, Midrand, South Africa) and the protocol was followed as per instructions.

### Western blotting

Briefly, cell lysates were prepared with 1× RIPA buffer and protein levels quantified via the BCA assay. Protein was then resolved on a 12% SDS/PAGE gel for 45–55 min at 150 V per gel. Subsequently, proteins were transferred onto a PVDF membrane using the Trans‐Blot^®^ Turbo™ Transfer system (Bio‐Rad, Hercules, California, USA). Membranes were thereafter blocked in 3% BSA in 1× PBS and 0.1% Tween 20 (PBST) for 1 h. After blocking, the blots were incubated with the anti‐LRP IgG1‐iS18 (Affimed Therapeutics, Heidelberg, Germany) and β‐actin (loading control, primary labelled anti‐β‐actin‐HRP – Cell Signaling Technology, Danvers, Massachusetts, USA) primary antibodies at a 1 : 10 000 dilution overnight with gentle shaking at 4 °C. The membranes were subsequently washed in PBST and the IgG1‐iS18 blots were further incubated for 1 h in the dark, in secondary antibody (anti‐Hu‐HRP – Abcam, Cambridge, UK) at a 1 : 10 000 dilution. The membranes were then washed as outlined above. The proteins were visualised with Clarity™ Western ECL Blotting Substrate and the ChemiDoc™ Imaging System (Bio‐Rad). Densitometric analysis was performed with image lab 5.1 software (Bio‐Rad), whereby all values were further normalised against the β‐actin loading control.

### MTT assay

Briefly, A549 cells were transfected as described. Protocatechuic acid (8 mm PCA; Sigma Aldrich) was used as a positive control. After incubation, 100 μL of 1 mg·mL^−1^ MTT was added to each well and incubated for 2 h at 37 °C. The resultant formazan crystals were dissolved in 200 μL DMSO per well and resuspended. The optical density was determined using a microplate reader at 570 nm. The percentage cell viability was then established by calculating the treated values as a percentage of the corresponding untreated values for each biological repeat, with the untreated set to 100%.

### Trypan blue exclusion

Briefly, cells were seeded and transfected as described. After a 72‐h transfection, the cells were resuspended in 20 μL Trypan blue dye (1 : 1; Gibco, Miami, Florida, USA) and measured with the TC 20™ automated cell counter (Bio‐Rad). The cell counter provided the percentage cell viability for each sample which was recorded and analysed statistically.

### Cell morphology

A549 cells were imaged 48‐ and 72 h post‐transfection using the Zeiss Primovert light microscope at 40× magnification. Images were then analysed visually for signs of morphological changes indicating apoptosis, including the appearance of cell shrinkage and membrane blebbing.

### APOPercentage™ cell apoptosis assay

The APOPercentage™ Cell apoptosis assay (Biocolor, Co Antrim, UK) was performed as per manufacturer's instructions. Briefly, cells were transfected and 30 min prior to the end of the 48‐ and 72‐h incubation periods, 5% v/v of the APOPercentage™ dye was added and incubated for 30 min at 37 °C. The dye‐containing medium was then removed and the cells were washed and imaged at 20× magnification. Subsequently, the cells were detached and APOPercentage™ Dye Release Agent was added. The absorbance was measured at 550 nm with a microplate reader. The fold increase was then calculated, where the untreated was set to 1.

### Scratch motility assay

The A549 cells were transfected and allowed to reach 90–95% confluency to ensure a confluent monolayer was created (approximately 24 h post‐transfection). A vertical scratch was made across the cell layer with a P2 pipette tip. A medium change was then performed containing 5 μg·mL^−1^ Mitomycin C (Sigma Aldrich) to inhibit cell proliferation. The wells were then imaged with the Zeiss Primovert light microscope (0 h) and were further imaged 24‐ and 48 h post‐scratch. Image analysis of the scratch was then performed using imagej, developed by National Institute of Health, USA and presented in terms of percentage wound closure.

### Telomerase activity assay

The TRAP assay was performed based on previous protocols [22] and modified in‐house. Briefly, the A549 cells were transfected with RPSA siRNA for 72‐h and harvested, cell pellets were subsequently resuspended in 200 μL of CHAPS (Merck, Darmstadt, Germany) Lysis Buffer/10^5^–10^6^ cells. The suspensions were incubated on ice for 30 min whereafter the samples were centrifuged at 12 000 × ***g*** for 20 min at 4 °C to pellet cell debris. The protein was then quantified with the NanoDrop™ One and standardised to 500 ng·μL^−1^ for all experimental and control reactions. The TRAP reaction mix was prepared and loaded into a 96‐well plate, together with 1 μg·μL^−1^ protein samples to a final volume of 12.5 μL. The reaction Mastermix consisted of Luna^®^ Universal qPCR Mastermix (New England Biolabs, Ipswitch, Massachusetts, USA), EGTA, TS, and ACX primers (Inqaba Biotec, Pretoria, South Africa), as well as Nuclease‐free (PCR grade) water. All samples were analysed via qPCR with the CFX96™ Touch qPCR System (Bio‐Rad) using the following cycling parameters: One cycle of 37 °C for 30 min, 95 °C for 2 min and 45 cycles of 95 °C for 15 s, 59 °C for 60 s and 45 °C for 10 s (where fluorescent reads were taken). Controls were included in the plate set up and included: a minus telomerase control, a no template control, a heat‐treated telomerase negative control and a telomerase positive cell extract as a positive control. The Cq values generated were then analysed using the Bio‐Rad CFX maestro software. All values were standardised to a percentage of the untreated controls which were set to 100%, after background subtraction of the heat‐treated samples. A schematic for this assay can be found in Fig. S1.

### Proteomics analysis

The A549 cells were seeded, transfected, and harvested as previously described. Cell pellets were snap frozen in liquid nitrogen and stored at −80 °C. Sample and data processing were performed (as described in [23]) at The Council for Scientific and Industrial Research (CSIR) centre, Pretoria, South Africa. Only proteins with a minimum fold change of ≥ 2 [24] and a maximum adjusted *P*‐value of ≤ 0.05 were considered to be differentially expressed. Protein set enrichment analysis was then further performed using the Search Tool for Retrieval of Interacting Genes/Proteins (STRING) database (v11.5) and the Reactome Pathway Browser tool to get an overview of the downstream effect on key pathways. STRINGdb (string‐db.org) is a biological database of known and predicted protein–protein interactions from numerous sources, including experimental data, public text collections, and computational prediction methods. Whereas the Reactome (reactome.org) Pathway Browser tool provides a means of viewing and interacting with pathways relating to a specific dataset, based on information within the Reactome database.

### Statistical analysis

Statistical analyses were performed using microsoft excel 365 (Microsoft Corporation, Redmond, Washington, USA) and graphpad prism Software (San Diego, California, USA). All experiments were performed with a minimum of three biological repeats with error bars representing standard deviation. A one‐way ANOVA analysis was performed, followed by pairwise comparisons using a two‐tailed student's *t*‐test, both at a 95% confidence interval; where *P* values < 0.05 were considered statistically significant (*P < 0.05, **P < 0.01 and ***P < 0.001).

## Results

Since downregulating LRP/LR with LRP mRNA‐targeting siRNA has been shown to have a significant therapeutic effect against various cancer types *in vitro*, this study included performing a transient transfection with LRP‐specific siRNA (RPSA siRNA) and subsequently evaluating the effect of the downregulation of LRP/LR on cell viability and apoptosis, migration potential, and telomerase activity. Further to this, a full proteomic analysis was performed to gain more insights into the role of LRP/LR in lung cancer, as well as an improved understanding into the pathways involved when LRP/LR is downregulated.

### siRNA‐mediated downregulation of LRP/LR decreases cell viability by inducing apoptosis

Resistance to cell death, including the evasion of apoptosis, is a crucial way in which cancer cells continue to survive, even when conditions are unfavourable for non‐cancerous cells. This is therefore a vital hallmark of cancer, which needs to be targeted as part of the search for cancer therapeutics. The overexpression of LRP/LR on cancer cells, significantly contributes towards the viability and inhibition of apoptotic pathways in cancer cells [25].

**Fig. 1:**
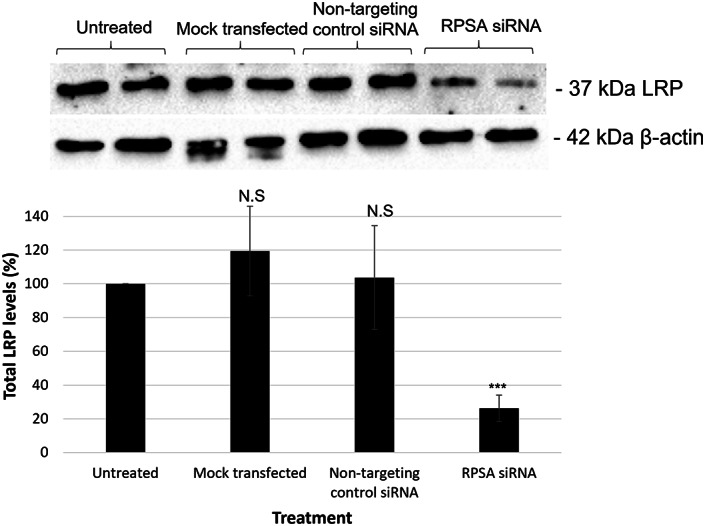
Transfection of A549 cells with LRP‐targeting siRNA significantly decreases total LRP/LR levels. Western blotting with subsequent densitometric analysis showed a significant decrease in LRP levels after a 72‐h transient transfection of A549 cells with the LRP‐specific siRNA (RPSA). Each bracket represents two biological repeats per treatment after 72 h transfection. One‐way ANOVA analysis showed a significant (**P = 0.0084) impact of the transfection on LRP levels, which were decreased by 74% in the RPSA siRNA transfected cells, with no significant effect on LRP levels observed in the NTC and mock transfected cells. β‐Actin was used as the loading control. the untreated sample is set to 100%, with all samples expressed as a percentage of the untreated samples. The data are representative of the mean ± SD, *n* = 3 biological repeats. *** ≤ *P*, 0.001; Student's *t*‐test.

**Fig. 2:**
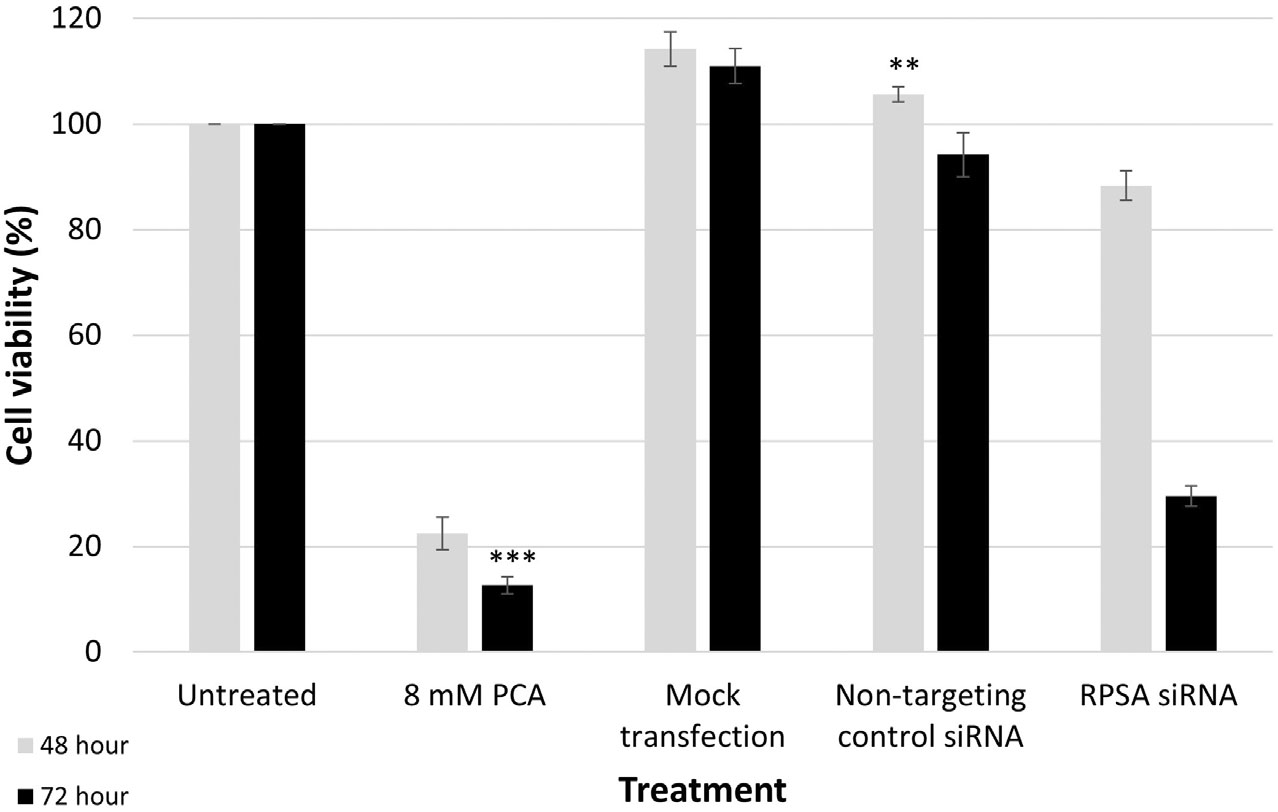
siRNA‐mediated downregulation of LRP/LR significantly decreases cell viability in A549 lung cancer cells. Cellular viability of A549 cells as determined by 3‐(4,5‐Dimethylthiazol‐2‐yl)‐2,5‐Diphenyltetrazolium bromide (MTT). Cell viability was assessed 48‐ and 72 h post‐transfection, with the untreated set to 100%. One‐way ANOVA analyses showed a significant impact of downregulating LRP/LR on decreasing cell viability after 48‐ (***P = 3.21E‐11) and 72 h (***P = 8.86E‐12), respectively. Protocatechuic acid (PCA) was used as a positive control. All values indicate the mean ± SD. Error bars represent standard deviation, *n* = 3 biological repeats. ** ≤ *P*, 0.01, *** ≤ *P*, 0.001; Student's *t*‐test.

We have previously shown that downregulating LRP/LR with siRNA‐LAMR1 reduced cell viability and induced apoptosis in A549 cells [17]. The current study served to confirm that these effects observed were due to LRP downregulation rather than off‐target effects specific to the siRNA‐LAMR1, and further served to elaborate upon these results by investigating the effects of LRP downregulation on other cancer hallmarks. Therefore, the present study used a different LRP‐targeting siRNA (esiRNA‐RPSA) and after successfully downregulating LRP (Fig. 1), cell viability was assessed via MTT and Trypan blue exclusion assays. A significant decrease in cell viability was observed in the A549 cells transfected with RPSA siRNA after both 48‐ and 72 h, whereas no significant decrease in cell viability was observed in the NTC siRNA and mock transfected controls when compared with the untreated cells (Fig. 2). In particular, when assessed via MTT assay, the cells transfected with RPSA siRNA for 48‐ and 72 h showed a significant 11.65% and 70.45% decrease in cell viability, respectively (Fig. 2). Additionally, when assessed via the Trypan blue exclusion assay for confirmation, the cells transfected with RPSA siRNA for 72 h showed that only 33.33% of the cells remained viable (Table 1). Therefore, confirming that indeed, the downregulation of LRP/LR significantly reduces cell viability.

**Table 1:** Cell viability was assessed via the trypan blue exclusion assay 72 h post‐transfection; indicating a significant effect of downregulating LRP/LR on cell viability.

Sample	Mean cell viability (*n* = 3)	*P*‐value (*T* test)
Untreated	98.33	N/A
Non‐targeting control siRNA	94	N.S 0.14576
RPSA‐siRNA	33.33	***0.00028

*** ≤ *P*, 0.001.

**Fig. 3:**
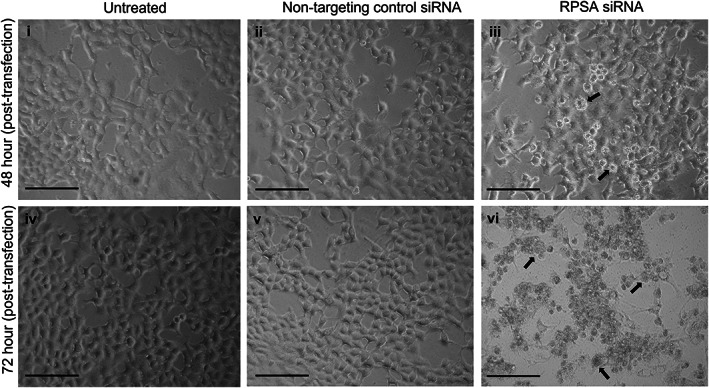
Downregulation of LRP/LR induces an apoptotic morphology in A549 cells. Light microscopy was used to assess the cellular morphological changes which occurred after downregulation of LRP/LR. Representative phase‐contrast images. (i–iii) A549 cells that were (i) untreated, (ii) transfected with the non‐targeting control (NTC) siRNA and (iii) transfected with RPSA siRNA and imaged 48 h post‐transfection. (iv–vi) A549 cells that were (iv) untreated, (v) transfected with the NTC siRNA and (vi) transfected with RPSA siRNA and imaged 72 h post‐transfection. Images i–ii and iv–v indicate the normal morphological characteristics of epithelial cells growing adherently in a monolayer, demonstrating that the NTC siRNA and transfection reagent have no effect on the morphology of the cells. (iii) A549 cells start to exhibit cell shrinkage and membrane blebbing suggestive of apoptosis after 48‐ h. (vi) after 72 h, most of the A549 cells are displaying apoptotic morphology, including cell shrinkage and membrane blebbing. Scale bars represent 100 μm and black arrows point to apoptotic cells. All images were taken at 40× magnification.

Cellular morphology was then assessed via light microscopy, and an APOPercentage™ assay was performed to confirm the mode of cell death occurring. Evident cell shrinkage and membrane blebbing, characteristic of apoptosis [26, 27], was observed after LRP/LR downregulation (Fig. 3). Untreated cells and cells transfected with NTC siRNA exhibited normal morphology with no indication of cell death observed both 48‐and 72 h post‐transfection (Fig. 3). Therefore, portraying that the downregulation of LRP/LR significantly decreases A549 lung cancer cell viability and induces an apoptotic morphology.

**Fig. 4:**
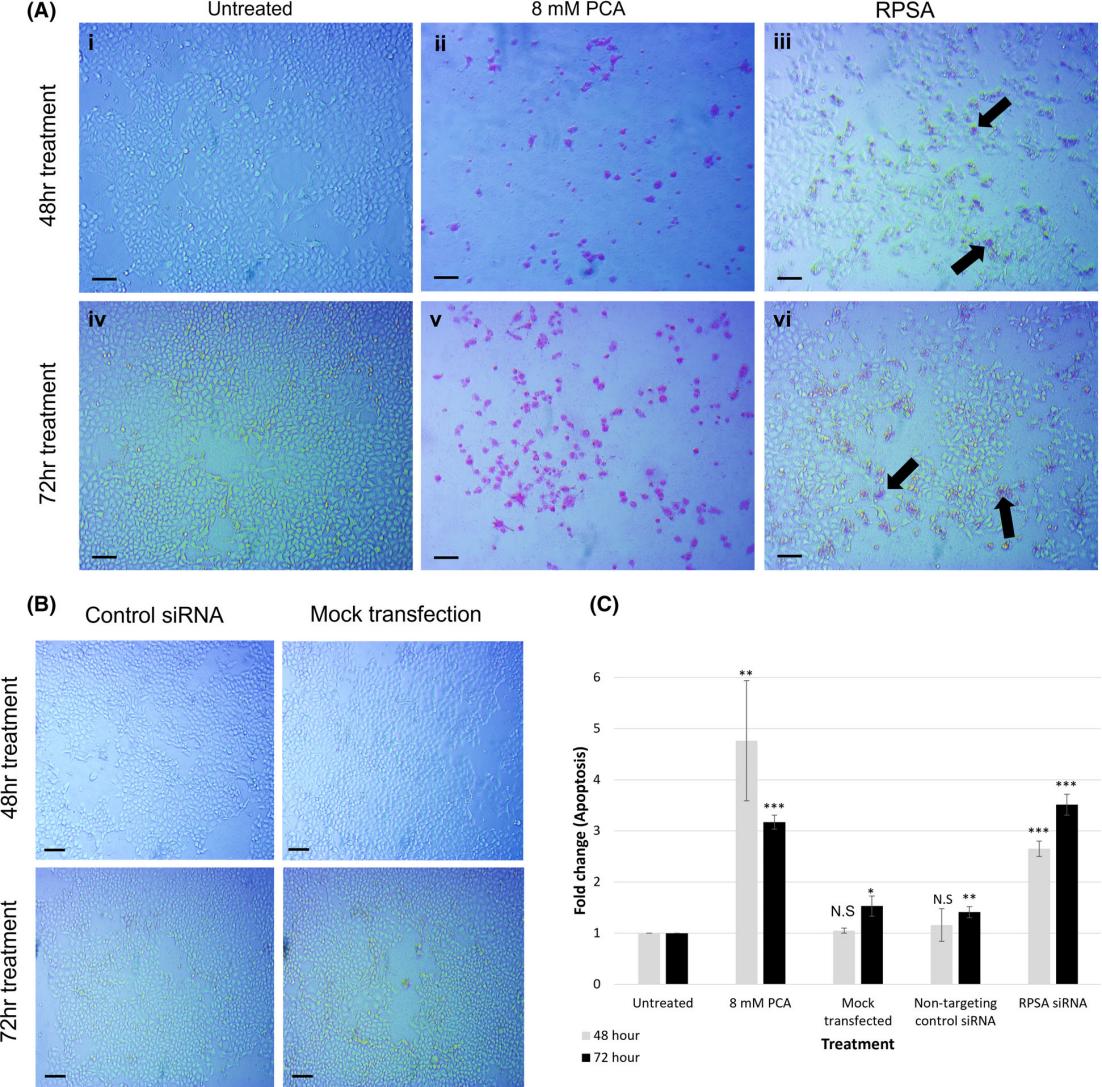
siRNA‐mediated downregulation of LRP/LR significantly increases apoptosis in A549 lung cancer cells. Apoptosis in A549 cells as determined by cell‐APOPercentage™ assay after downregulation of LRP/LR with RPSA siRNA. (A) Representative phase‐contrast images of the A549 cells 48‐ and 72 h post‐transfection, with pink cells stained with the APOPercentage™ dye undergoing apoptosis. (i–iii) A549 cells (i) untreated, (ii) treated with 8 mM PCA and (iii) transfected with RPSA siRNA for 48 h, indicating the occurrence of apoptosis in the PCA treated and RPSA siRNA transfected cells compared with the untreated cells displaying no apoptosis. (iv–vi) A549 cells (iv) untreated, (v) treated with 8 mM PCA and (vi) transfected with RPSA siRNA for 72 h indicating the occurrence of apoptosis in the PCA treated and RPSA siRNA transfected cells. (B) Representative images of the NTC and mock transfection 48‐ and 72 h post‐transfection. (C) Colorimetric analysis of the fold change in apoptosis was performed 48‐ and 72 h post‐transfection. One‐way ANOVA analysis showed a significant effect of the treatments on apoptosis after 48 (***P = 2.73E‐05) and 72 (***P = 2.47E‐08) hours. The untreated sample was set to 1 and 8 mm protocatechuic acid (PCA) was used as a positive control. the mock transfected and cells transfected with non‐targeting control (NTC) siRNA^1^ present no significant increase in apoptosis. Scale bars represent 100 μm. All values indicate the mean ± SD, *n* = 3 biological repeats. * ≤ *P*, 0.05, ** ≤ *P*, 0.01, *** ≤ *P*, 0.001; Student's *t*‐test.

Subsequently, the APOPercentage™ assay showed a significant 1.65‐ and 2.51‐fold increase in apoptosis in the A549 cells transfected with RPSA siRNA after 48‐ and 72 h, respectively (Fig. 4B). This therefore indicates that indeed the decrease in cell viability observed after downregulation of LRP/LR can be attributed to apoptosis, confirming our previous results with the siRNA‐LAMR1 [17].

Furthermore, this effect is certainly time‐dependent as observed by the small, nonetheless significant, effects seen in cell viability (11% decrease; Fig. 2) and apoptotic morphology (Fig. 3), as well as apoptotic induction (1.65‐fold; Fig. 4B) 48 h post‐transfection compared with those at 72 h as described above. This exponential increase in the effect observed at 72 h versus 48 h is indicative that once LRP levels have decreased below a certain level, the cells are no longer able to compensate for this, and the effect of LRP knockdown becomes detrimental to the entire cell population. Which is an outcome that shows potential for targeting entire tumours. The 72‐h transfection was therefore the time point chosen for assessment of the effect of LRP downregulation on telomerase activity as well as for proteome analysis, as this represented the late‐stage effects.

### Downregulating LRP/LR decreases cell migration potential

**Fig. 5:**
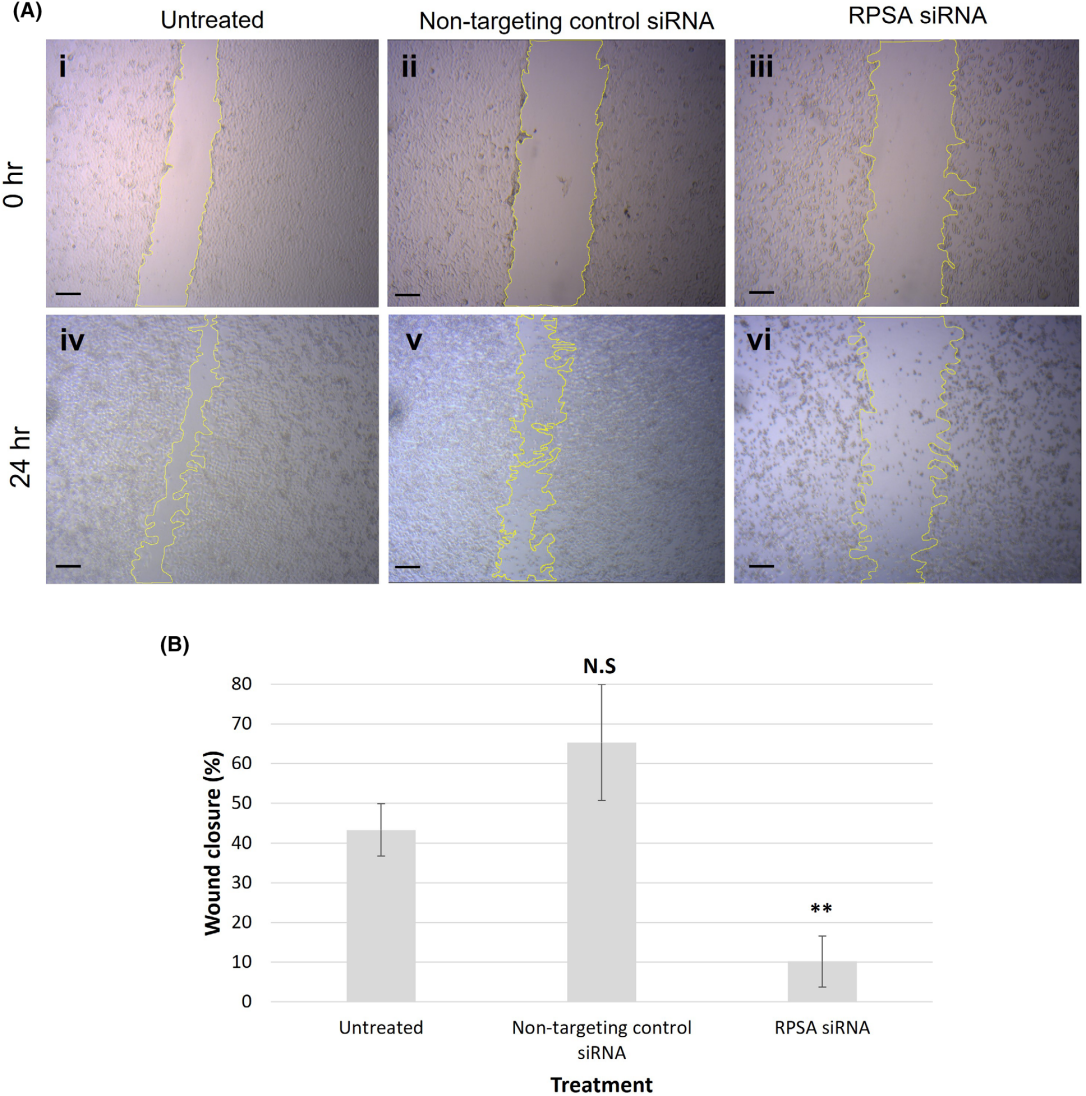
Downregulation of LRP/LR significantly decreases A549 cellular migration. A scratch motility assay shows a significant decrease in the percentage wound closure, and therefore, a significant decrease in the migration potential of A549 cells after 48‐h siRNA‐mediated downregulation (RPSA siRNA) of LRP/LR. A549 cells were transfected 24 h prior to the scratch assay being performed, with images taken 24 h (0 h, when the scratch was made) and 48 h (24 h post‐scratch) post‐transfection. (A) Representative phase‐contrast images show the decrease in cell migration, whereby the yellow outlines indicate the area of the wound. (i–iii) A549 cells (i) untreated, (ii) transfected with non‐targeting control (NTC) siRNA and (iii) transfected with RPSA siRNA for 24 h. (iv–vi) A549 cells (iv) untreated, (v) transfected with NTC siRNA and (vi) transfected with RPSA siRNA for 48 h, and imaged 24 h post‐scratch, showing varying degrees of cell migration has occurred. (B). Analysis of the difference between the area of the scratch at 0 h and at 24 h post‐scratch using imagej software, indicates a significant decrease (**P = 0.0015, one‐way ANOVA analysis) in wound closure, and therefore, migration potential in the RPSA transfected A549 cells, while the non‐targeting control (NTC) siRNA has no significant effect on migration potential. Scale bars represent 100 μm. The data are representative of the mean ± SD, *n* = 3 biological repeats. N.S., non‐significant, ** ≤ *P*, 0.01; Student's *t*‐test.

Cell migration is a key process in metastasis, therefore, the effect of LRP downregulation on the migration potential of the A549 cells was assessed via a scratch assay (Fig. 5). After 24 h of transfection the scratch was made, and the cells imaged immediately (0 h; Fig. 5A.i–iii). The cells were subsequently imaged 24 h post‐scratch, thus 48 h after transfection (Fig. 5A.iv–vi). The scratch assay was analysed 48 h post‐transfection since previous assays, as described above, had shown the downstream effects of LRP downregulation were visible, but minimally at 48 h post‐transfection. However, as the cells are only ±30% viable at 72 h post‐transfection, we were not able to accurately observe the effect on cell migration at this time‐point (data not shown).

The cells transfected with the RPSA siRNA exhibited only 10.17% wound closure, compared with the untreated cells exhibiting 42.29% wound closure 48 h after transfection (Fig. 5B). No significant difference in wound closure was observed in the NTC siRNA transfected cells, when compared with the untreated cells.

### Downregulating LRP/LR suppresses telomerase activity

**Fig. 6:**
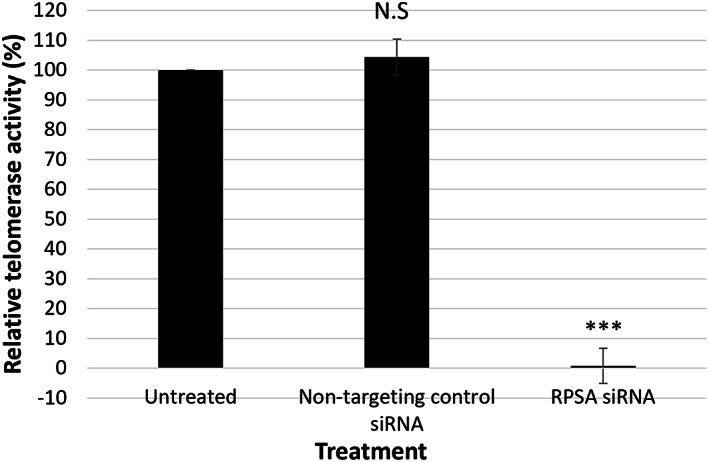
Downregulation of LRP/LR significantly decreases telomerase activity in A549 cells. Relative telomerase activity is significantly (***P = 1.23E‐04) affected in A549 cells after 72‐h transfection, as shown by one‐way ANOVA analysis. All values were normalised against the negative telomerase activity/heat‐treated controls. LRP‐specific (RPSA) siRNA downregulation of LRP/LR caused a significant 99.19% decrease in telomerase activity. The cells transfected with the NTC siRNA exhibited no significant change in telomerase activity. The untreated sample is set to 100%, with all samples expressed as a percentage of the untreated samples. The data are representative of the mean ± SD, *n* = 3 biological repeats. N.S., non‐significant, *** ≤ *P*, 0.001; Student's *t*‐test.

The upregulation of telomerase activity in cancer cells is an additional way in which cancer cells tend to override inhibitory signals, which enables replicative immortality [15]. To determine if downregulation of LRP affects telomerase activity, the modified TRAP assay was performed via qPCR. A highly significant 99.19% decrease in telomerase activity was observed in the RPSA siRNA transfected cells (Fig. 6). The NTC siRNA transfected samples showed no significant change in telomerase activity. Thus, the downregulation of LRP/LR almost completely inhibits telomerase activity in the A549 lung cancer cells, and as such, targets the ability for replicative immortality, a hallmark of cancer.

### Assessing the effect of downregulating LRP/LR on the proteome of A549 cells

To get a broader overview of the mechaonistic role of LRP/LR downregulation, SWATH‐MS was performed after a 72‐h transfection with RPSA siRNA, with subsequent protein set enrichment analysis using STRING database and the Reactome pathway browser. For the full list of details regarding the dysregulated proteins and their function, refer to Tables S1–S3.

**Fig. 7:**
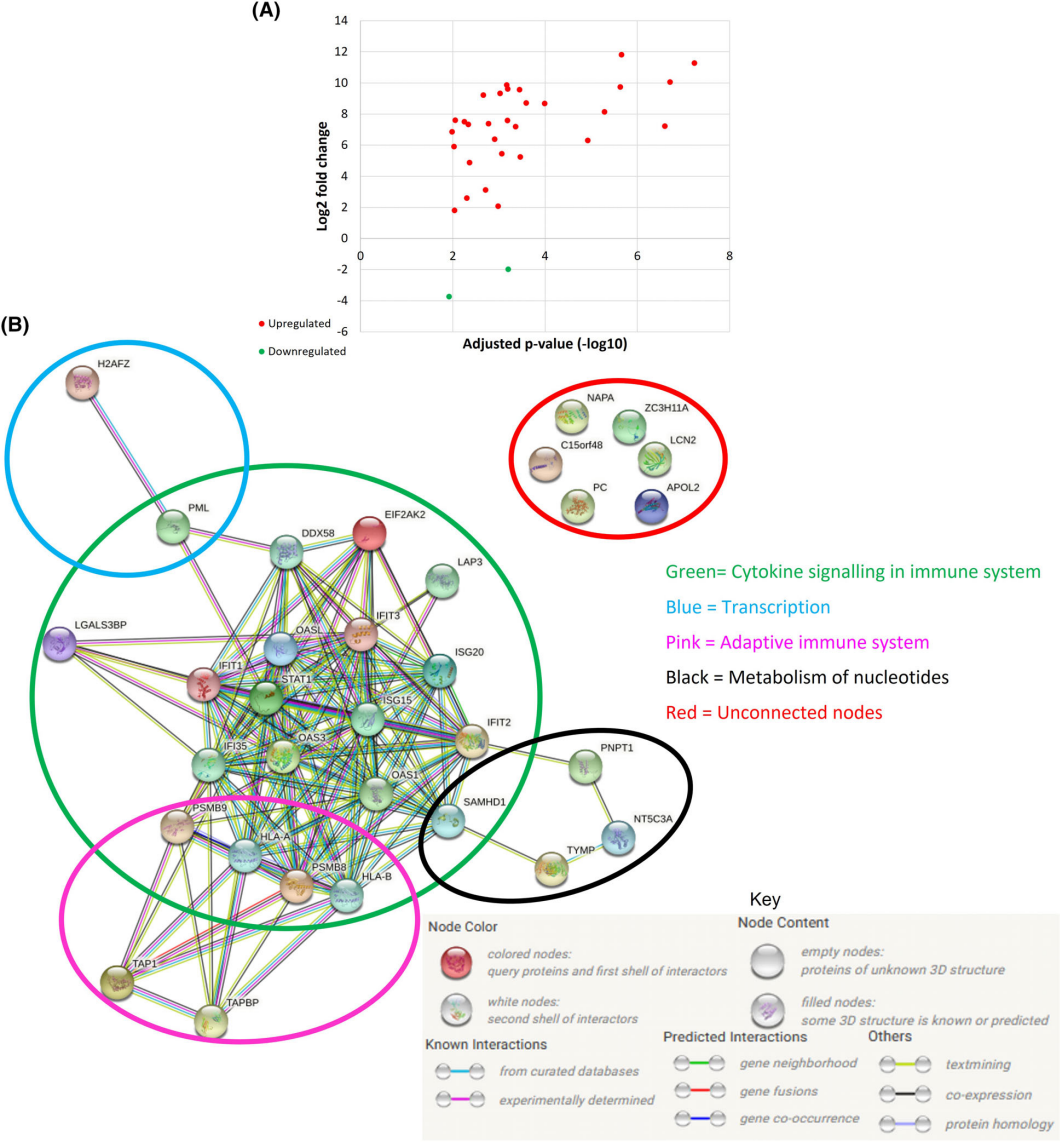
Identification of interactions between all dysregulated proteins above a two‐fold change after LRP downregulation and analysed via STRING database. Protein–protein interaction network of all dysregulated proteins with a fold change of ≥ 2 associated with downregulation of LRP/LR after a 72‐h transfection. (A) a scatter plot indicating the dysregulated proteins identified via SWATH‐MS; 30 proteins were upregulated, and two proteins were downregulated. (B) Protein–protein network of all dysregulated proteins with a fold change of two or above, four distinct protein–protein interaction networks are clustered according to common shared pathways. These include the main cluster (green) involved in cytokine signalling in the immune system, a cluster of six proteins (pink) involved in the adaptive immune system, a cluster of four proteins (black) involved in nucleotide metabolism, a cluster of two peptides (blue) involved in transcription, as well as a cluster of 6 unconnected proteins (red) which indicate a lack of interaction with any of the other query proteins. PPI enrichment *P*‐value: < 1.0E‐16.

**Fig. 8:**
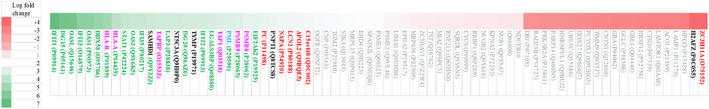
Heat map of the dysregulated proteins after LRP downregulation. The heat map shows the dysregulated proteins and their various fold changes with the respective protein names shown in different colours representing the four distinct common shared pathways shown in the protein–protein interaction network. The proteins in green are involved in cytokine signalling in the immune system, proteins in pink are involved in the adaptive immune system, proteins in black are involved in nucleotide metabolism, protein names in blue are involved in transcription, as well as the six unconnected proteins represented in red.

All the dysregulated (both up‐and downregulated) proteins identified to have a fold change of 2 and above were input to STRINGdb, which provided valuable insight into the interactions occurring amongst the dysregulated proteins (Fig. 7). Thereafter, these proteins were further analysed with the Reactome Pathway Browser tool, which then overlaid this supplied dataset to the pathways identified by Reactome. However, the output from Reactome provides all possible pathways in which the proteins have been previously identified to be involved in, which have been highlighted in Fig. 8, Table 2, and Table S1. Thus, using STRINGdb to identify the protein interactions, combined with the identification of possibly involved pathways using Reactome, allowed us to cluster the proteins with a fold change of 2 and above according to the commonly shared pathways, and therefore infer the possible mechanism occurring. In so doing, 28 out of the 32 dysregulated proteins that were identified with a 2‐fold change and above were found in Reactome, where 320 pathways were hit by at least one of the query proteins. Furthermore, four distinct protein––protein interaction networks were identified and clustered according to common shared pathways, some of which overlap (Fig. 7). These include the main cluster of 21 peptides involved in cytokine signalling in the immune system, a group of six proteins each involved in the adaptive and innate immune systems, respectively. At least one of each of the remaining proteins were found to play a role in various other pathways including transcription, nucleotide and protein metabolism, cell cycle checkpoints, apoptosis, signal transduction, and transport of small molecules via vesicle‐mediated transport. The protein–protein interaction enrichment *P*‐value was calculated by STRINGdb to be less than 1.0E‐16, which suggests that the query proteins have more interactions amongst themselves than what would be projected for a random set of proteins of the same size and degree distribution drawn from the genome. This enrichment therefore indicates that this group of proteins are at least moderately biologically associated. The full list of pathways identified by Reactome for each of these proteins can be found in Table S1.

**Table 2:** List of protein identifiers and protein names shown in the STRING network.

STRING identifier (UniProt ID)	Protein name
Downregulated	
ZC3H11A (O75152)	Zinc finger CCCH domain‐containing protein 11A
H2AFZ (P0C0S5)	Histone H2A.Z
Upregulated	
C15orf48 (Q9C002)	Normal mucosa of oesophagus‐specific gene 1 protein
APOL2 (Q9BQE5)	Apolipoprotein L2
LCN2 (P80188)	Neutrophil gelatinase‐associated lipocalin
NAPA (P54920)	Alpha‐soluble NSF attachment protein
PNPT1 (Q8TCS8)	Polyribonucleotide nucleotidyltransferase 1, mitochondrial
PC (P11498)	Pyruvate carboxylase, mitochondrial
EIF2AK2 (P19525)	Interferon‐induced, double‐stranded RNA‐activated protein kinase
PSMB8 (P28062)	Proteasome subunit beta type‐8
PSMB9 (P28065)	Proteasome subunit beta type‐9
PML (P29590)	Protein PML
TAP1 (Q03518)	Antigen peptide transporter 1
LGALS3BP (Q08380)	Galectin‐3‐binding protein
IFIT2 (P09913)	Interferon‐induced protein with tetratricopeptide repeats 2
TYMP (P19971)	Thymidine phosphorylase
ISG20 (Q96AZ6)	Interferon‐stimulated gene 20 kDa protein
NT5C3A (Q9H0P0)	Cytosolic 5′‐nucleotidase 3A
LAP3 (P28838)	Cytosol aminopeptidase
TAPBP (O15533)	Tapasin
SAMHD1 (Q9Y3Z3)	Deoxynucleoside triphosphate triphosphohydrolase
IFI35 (P80217)	Interferon‐induced 35 kDa protein
OAS3 (Q9Y6K5)	2′–5′‐oligoadenylate synthase 3
STAT1 (P42224)	Signal transducer and activator of transcription 1
HLA‐A (P04439)	HLA class I histocompatibility antigen, A‐3 alpha chain
HLA‐B (P01889)	HLA class I histocompatibility antigen, B‐7 alpha chain
DDX58 (O95786)	Probable ATP‐dependent RNA helicase
OAS1 (P00973)	2′–5′‐oligoadenylate synthase 1
IFIT3 (O14879)	Interferon‐induced protein with tetratricopeptide repeats 3
OASL (Q15646)	2′–5′‐oligoadenylate synthase‐like protein
ISG15 (P05161)	Ubiquitin‐like protein ISG15
IFIT1 (P09914)	Interferon‐induced protein with tetratricopeptide repeats 1

## Discussion

The global occurrence and mortality rates of cancer are growing at an exponential rate. In particular, lung cancer is the most frequently occurring cancer and the foremost cause of cancer‐related deaths. The fundamental cause of the extremely complex, multi‐step process of tumourigenesis are the hallmarks of cancer and their enabling characteristics. Since there is co‐dependence and complementarity amongst these hallmarks, prospective treatments need to consider targeting them on multiple levels, rather than independently. The 37 kDa laminin receptor precursor/67 kDa laminin receptor (LRP/LR) is overexpressed in many different cancer types, and as a result is involved in promoting the tumourigenic process by its involvement in enhancing cell migration, adhesion and invasion, maintaining cell viability, inducing angiogenesis and evading apoptosis, as well as increasing telomerase activity. The current study revealed that siRNA‐mediated downregulation of LRP/LR in A549 lung cancer cells significantly inhibits the characteristics of multiple cancer hallmarks.

### Downregulation of LRP/LR decreases cell viability by inducing apoptosis

A vital hallmark of cancer includes the resistance of cancer cells to cell death processes, which includes apoptotic evasion and is a crucial way in which cancer cells continue to survive under conditions where normal cells would undergo apoptosis [3, 4]. This is therefore a common focus as part of the search for cancer therapeutics. The overexpression of LRP/LR on cancer cells significantly contributes towards cell viability and inhibition of apoptotic pathways in cancer cells [6]. Thus, studies involving the downregulation of LRP/LR have placed emphasis on the subsequent effect on cell viability. Various studies have confirmed that siRNA‐mediated downregulation of LRP/LR in cancer cells overexpressing LRP/LR, results in apoptotic induction through different pathways, depending on the cancer cell type. In particular, apoptosis was induced in breast, oesophageal [28], cervical, and lung [17] cancer cells after LRP downregulation. Additionally, other studies showed that caspase −8 and −9 are activated after knockdown of LRP/LR in pancreatic and neuroblastoma cancer cells [18], in early and late‐stage colorectal cancer cells [20], as well as in early and late‐stage malignant melanoma cells [19]. Since there was a significant decrease in cell viability previously observed in A549 cells after siRNA‐LAMR1‐mediated downregulation of LRP/LR [17], the current study included an investigation into the effect on cell survival, migration, and the proteome after RPSA siRNA mediated downregulation of LRP/LR in the A549 cells. A significant decrease in cell viability was observed in the A549 cells transfected with RPSA siRNA after both 48‐ and 72 h as previously observed and it was confirmed that the mode of cell death induced was apoptosis. Therefore, the current study showing the induction of apoptosis in the A549 cells using a different LRP‐targeting siRNA confirms that indeed the effects observed were occurring due to the downregulation of LRP and not due to off‐target effects. This further confirms the role of downregulating LRP in inhibiting the cancer hallmark, ‘Resisting cell death’.

### LRP/LR downregulation decreases migration potential

Metastasis is the primary cause of poor prognoses and cancer lethality, whereby 90% of deaths caused by solid tumours are attributed to metastatic propagation. Understanding the multi‐step process of migration, adhesion, and invasion for each individual cancer type is a mammoth task [29], but one which is absolutely necessary in the search for therapeutic intervention. Therefore, a fundamental factor for consideration when developing novel treatment strategies for cancer is certainly determining the effect of the treatment on cell migration, adhesion, and invasion [30]. Furthermore, LRP/LR has been implicated in the metastatic process through its interaction with laminin‐1, whereby the overexpression of LRP/LR on cancer cells increases this interaction, causing enhanced migration and invasion of the tumourigenic cells [31, 32]. A crucial tool when investigating the therapeutic potential of novel anti‐cancer treatments, is the quantification of *in vitro* migration processes in cancer cell lines by means of time‐lapse microscopy. Which can further provide a basic understanding of the migration‐related pathways involved in the metastatic processes of that particular cancer type *in vivo*. Thus, a scratch motility assay was performed after RPSA siRNA transfection to determine whether LRP downregulation would affect the migration potential of the A549 lung cancer cells.

There was a significant decrease of 32.12% observed in migration potential in the A549 cells after LRP downregulation. Furthermore, since the anti‐proliferative agent, Mitomycin C [33] was included during the scratch assay, it can confidently be concluded that the wound closure observed was due to cell migration, rather than cell proliferation. The core part of the enhanced interaction between laminin‐1 and LRP in cancer cells overexpressing LRP, is that it causes the release of type IV collagenase, which degrades the basal lamina, thereby allowing the cells to migrate through the blood stream to distant sites [31, 32]. Previous studies have shown that downregulating LRP/LR decreases the adhesive and invasive potential of tumourigenic fibrosarcoma (HT1080) cells overexpressing LRP/LR [34]. Altogether, there is strong evidence showing the role of downregulating LRP/LR to reduce metastatic processes. Therefore, we suggest that the downregulation of LRP/LR reduces the migration potential of the cells, and thereby plays a role in inhibiting the ‘Activating invasion and metastasis’ cancer hallmark.

### Downregulation of LRP/LR inhibits telomerase activity

Not only is the upregulation of telomerase activity another way in which cancer cells supersede inhibitory signals, but high telomerase activity levels are also associated with cancer recurrence and chemotherapeutic resistance [35]. Accordingly, telomerase activity has been detected in 75% of NSCLC [36]. We have previously demonstrated that LRP levels are directly associated with telomerase activity, in that LRP overexpression significantly increases the TERT levels and telomerase activity [10, 12], and the downregulation of LRP/LR significantly decreases the telomerase activity in breast cancer cells [9, 23].

Therefore, the modified TRAP assay was performed via qPCR to detect the telomerase activity levels after the A549 cells were transfected with RPSA siRNA for 72 h. Interestingly, the downregulation of LRP/LR caused a consequent, highly significant decrease in telomerase activity, whereby the cells transfected with RPSA siRNA displayed a 99.19% decrease in telomerase activity. Thus, the downregulation of LRP/LR causes an almost complete inhibition of telomerase activity in the A549 lung cancer cells, and as such, targets the cells' ability for replicative immortality, therefore referring to the inhibition of the ‘Enabling replicative immortality’ cancer hallmark.

It is well‐known that cancer cells are able to avoid the effects of senescence and apoptosis through the upregulation of telomerase activity. This thereafter permits the cells to maintain unstable telomere lengths [37], which stabilises the mutated genome and enables replicative immortality [4], whereafter macroscopic tumours are formed [3]. The downregulation or inhibition of these higher levels of telomerase activity has thus become a popular target for potential cancer therapeutics. Moreover, since telomerase activity is undetectable in most human somatic cells [38], targeting the upregulated telomerase levels in cancer cells or tumours should have few off‐target effects. A previous study has shown that targeting hTERT causes a decrease in the telomerase activity in lung cancer cells, which caused the cells to enter senescence via a p53‐dependent mechanism [39]. Another study showed that by targeting TERT levels directly with siRNA in A549 lung cancer cells, telomerase activity was reduced. They further showed *in vivo* that the downregulation of TERT via siRNA reduced the size of lung xenograft tumours due to apoptotic induction [36]. Additionally, this study performed by Xie et al. [36], demonstrated a complete inhibition of telomerase activity after 72‐h transfection that was causally associated with a 95% reduction in TERT levels at the same time‐point, which corresponds to the knockout of telomerase activity observed in the current study at 72 h post‐transfection. Interestingly, there was also a marked increase in apoptosis observed after telomerase inhibition. Perhaps more pertinently to this study, it has very recently been demonstrated that siRNA‐mediated downregulation of LRP in colorectal cancer cells, not only decreased the telomerase activity, but also decreased the TERT and phosphorylated TERT (pTERT) levels. Here, pTERT is the activated form of TERT, which specifically functions as the catalytic subunit in telomerase activity [23]. Furthermore, we propose that not only does this inhibition of telomerase activity reduce cell proliferation, but it also contributes to the increase in apoptosis observed in the present study. In particular, a study has shown that telomerase repression, either through inactivation of TERT or through shRNA inhibition of telomerase, increases cell cycle arrest and apoptosis in both *in vitro* and *in vivo* cancer models [40]. In addition, this decrease in telomerase caused an increase in expression of the promyelocytic leukaemia protein (PML), resulting in its translocation to the nucleus, with subsequent PML‐dependent recruitment of p53 into the nucleus and co‐localisation between the two resulting in the activation of p21 (to inhibit cell cycle progression) [40]. Thus, the inhibition of telomerase resulted in the activation of the PML‐dependent p53 pathway to induce cell cycle arrest and apoptosis. Interestingly, the downregulation of LRP/LR in the present study not only inhibited telomerase, but also caused the upregulation of PML (described below). Therefore, we suggest that the likely mechanism by which the observed decrease in cell viability and induction of apoptosis is occurring in the present study, is because the downregulation of LRP subsequently inhibits telomerase, which consequently activates PML‐dependent p53 signalling. We did not observe a change in p53 levels during proteomic analysis; however, the nuclear translocation and activation of p53 does not necessarily mean that the levels of p53 are affected.

### LRP/LR downregulation causes differential changes in protein levels

Proteomic analysis revealed the upregulation of numerous proteins involved in re‐establishing immune response, inhibiting cell proliferation, and inducing apoptosis, as well as in improving therapeutic response after LRP downregulation.

#### Upregulated proteins in re‐establishing immune response

Various upregulated proteins identified after downregulation of LRP/LR appear to play a role in the MHC I antigen presentation pathway. In particular, PSMB8 and PSMB9 were upregulated and are proteasomal subunits involved in generating class I binding peptides through antigen processing. TAP1(TAP) plays a role in transporting antigens from the cytoplasm to the ER for association with the MHC I molecules, while TAPBP (Tapasin) is part of the complex responsible for loading the peptide onto the MHC I molecules [41], both of which were found to be upregulated. Another protein found to be upregulated was NAPA. Reactome showed a role of NAPA in vesicular transport between the ER and Golgi apparatus. Therefore, we suggest NAPA is involved in this pathway by possibly transporting the peptide‐loaded MHC I molecules from the ER to the Golgi. Furthermore, both HLA‐A and HLA‐B were found to be significantly upregulated, and these proteins are the core subunits of the MHC I molecules [42]. The MHC I antigen presentation pathway is the mechanism which allows CD8+ T cells to identify cells producing foreign proteins, such as from mutant genes in cancers [43]. Therefore, cancer cells that have defects in various places within this pathway, including making, transporting, and loading MHC I molecules, become less visible to the immune system, thus impairing immune control of the tumours by CD8+ T cells [43]. Indeed, various cancer types lose expression of MHC I molecules through a variety of different ways including, but not limited to, mutation or deletion of genes involved, transcriptional regulation, posttranslational modification, and extrinsic stimuli from the tumour microenvironment. In this context, the loss of this pathway is a crucial way in which cancers are able to evade immune control. Furthermore, the loss of this pathway is not essential for cell viability or growth and is therefore not detrimental to the cancer cells. This is of clinical significance, in that the loss of this pathway is associated with poor prognosis and worse clinical outcomes [44, 45]. Interestingly, the STAT1 transcription factor was also observed to be upregulated and has been shown to be involved in the activation of the MHC I antigen presentation pathway. Although there was an observed increase in STAT1 protein levels, we do not have clarity on whether this is in its activated (pSTAT1) form. However, activated STAT1 is known to translocate to the nucleus and induce expression of TAP1 and indeed we observed an upregulation in TAP1 expression, as described above. It is therefore highly likely that, in the present study, the downregulation of LRP enhanced STAT1 expression, which activated transcription of the observed upregulated proteins in the MHC I pathway [46]. Ultimately, we propose a novel function of LRP/LR in cancer immune evasion, whereby the downregulation of LRP/LR activates the MHC I antigen presentation pathway for detection of the cancer cells by CD8+ T cells. Thus, targeting LRP/LR for downregulation is possibly re‐establishing immune control over the cells, thereby inhibiting an additional cancer hallmark referred to as ‘Evading immune destruction’ [4]. It would be interesting indeed, to be able to assess this *in vivo*, where the tumour microenvironment and immune system are able to act on the tumour cells.

#### Dysregulated proteins in inhibiting cell proliferation and inducing apoptosis

Most of the upregulated proteins identified after downregulation of LRP/LR have been shown to play important roles in inhibiting tumourigenesis via inhibiting cell proliferation and inducing apoptosis. This included ISG15, a ubiquitin‐like protein serving as a type of lung cancer tumour suppressor [47, 48, 49, 50, 51] and PML, a key component of PML nuclear bodies (PML‐NBs), which plays the role of a tumour suppressor by contributing to apoptotic induction, inhibiting angiogenesis as well as cell cycle progression [52, 53, 54]. PML is additionally involved in checkpoint response to DNA damage, whereby it has a function in inducing G1‐arrest and/or DNA repair (reviewed in [55]). In addition, the PML‐NB‐associated proteins, ISG20, OAS3, OAS1, and OASL were found to be upregulated and are considered to be tumour suppressors involved in promoting DNA cleavage, nuclear condensation, and apoptosis [56, 57, 58]. Furthermore, DDX58 protein levels were increased and has been seen to play a role alongside OASL, whereby downregulation of these proteins is observed to enhance cancer cell motility and invasion [59]. In addition, EIF2AK2 (often also referred to as PKR) is a double‐stranded RNA‐activated protein kinase, which has additionally been reported to suppress tumour growth [60], which was upregulated after LRP downregulation. Furthermore, analysis of NSCLC patient primary tissues showed not only a reduction in PKR, but also in OAS, further portraying their roles as lung cancer tumour suppressors [61]. H2A.z is upregulated in NSCLC which amplifies cell growth and survival [62]. We observed a downregulation in H2A.z after LRP downregulation, which suggests a reduction in the expression of cell cycle proteins that again correlates with the reduction in cell viability that was observed in the A549 cells.

Furthermore, as mentioned above, the STAT1 transcription factor was shown to be upregulated. Increased STAT1 expression has been fundamentally correlated with tumour suppression. The loss of STAT1 has been observed in lung cancer tumours and cancer patients exhibiting high STAT1 expression tend to have better clinical outcomes. STAT1 carries out its tumour suppressor role by inducing gene expression of proteins involved in cell growth arrest, apoptosis [63], and angiogenic inhibition [64] (reviewed in [65]). SAMHD1, a dNTP hydrolase, was also found to be upregulated. It is involved in reducing dNTP concentration and therefore plays a role in inhibiting cell proliferation and tumourigenesis [66]. Indeed, overexpression of SAMHD1 has been found to reduce the proliferation of A549 lung cancer cells [67].

Therefore, based on the significant upregulation of these proteins, as well as their functions, there is a clear role in immune presentation, cell cycle modulation and apoptosis, which corresponds with the reduction in cell viability and apoptotic induction as observed in the present study. We also speculate that since the cells are mostly apoptotic after a 72‐h transfection with RPSA siRNA, the immune and proteasomal degradation systems are being activated to clear the apoptotic cells and any resulting debris to regulate the cell death process and avoid tumour‐promoting inflammation. This is further suggested by the lack of dysregulated proteins identified in pro‐inflammatory pathways, indicating no apparent inflammatory response.

#### Dysregulated proteins involved in improving therapeutic response

The interferon‐induced protein with tetratricopeptide repeats (IFIT) gene family form part of the interferon‐stimulating genes (ISGs) and in the current study, several IFIT proteins (IFIT1, IFIT2 IFIT3 and IFI35) were found to be upregulated after LRP downregulation. Recent studies into the role of the IFIT family of proteins have shown an anti‐oncogenic function, aside from their role in host immunity and viral response [68]. In particular, increased IFIT1 and IFIT3 expression has been correlated with improved therapeutic response to chemotherapeutics and immunostimulating agents [69, 70] and increased IFIT2 expression in the tumour tissues of patients was associated with improved patient survival [71]. IFI35 is considered a radiotherapy‐associated gene [72] and is associated with cellular immune responses, including in the proteasome accessory complex in lung adenocarcinoma [73]. It has been shown that the IFIT proteins are activated by STAT1 [74], therefore, it appears that the downregulation of LRP induces activation of STAT1, which is then able to activate several of the proteins that have been observed to be upregulated.

Furthermore, TYMP and NT5C3A are important enzymes forming part of the pyrimidine salvage pathway [75], which is crucial in converting 5′ deoxy‐5‐fluorocytidine (5’DFCR) to 5‐fluorouracil, a key chemotherapeutic agent for treating solid tumours [76]. Therefore, the downregulation of LRP has also led to the upregulation of proteins known to be involved in sensitising cancer cells to current chemotherapeutics and radiotherapy. It is therefore possible that targeting LRP/LR for downregulation could be used as a means of enhancing therapeutic response to these existing treatment strategies to perhaps improve the clinical outcome for patients.

## Conclusion

In conclusion, this study reveals siRNA‐mediated downregulation of LRP/LR inhibits several key cancer hallmarks in lung cancer cells including *Resistance to cell death*, *Activation of metastasis*, *Enabling replicative immortality*, and *Evasion of immune destruction*. Therefore, this strategy of targeting LRP/LR has a 4‐fold function in impeding key cancer hallmarks in the lung cancer cells, which therefore has the potential for improving the prognosis of those suffering from lung cancer. Altogether, we suggest targeting LRP/LR for downregulation provides a possibly powerful therapeutic for inhibiting multiple cancer hallmarks.

## Conflict of interest

The authors declare no conflict of interest.

## Author contributions

MJB involved in conceptualisation, data curation, acquisition and analysis, and writing of original draft. TCO involved in review and editing of the manuscript. MB involved in assistance with experimental procedures. EF involved in conceptualisation, funding acquisition, supervision and editing of the manuscript; SFTW involved in conceptualisation, funding acquisition and supervision.

## Supporting information


**Fig. S1.** Modified Telomeric Repeat Amplification Protocol for real‐time quantification of telomerase activity. This protocol makes use of fluorescent dsDNA binding dyes which allows for detection and quantification of telomerase activity by directly measuring real‐time fluorescence emission via qPCR. Step 1 (Extension) of the reaction allows the telomerase enzyme in an extracted sample to add telomeric repeats to the 3′ end of a Telomeric Substrate (TS). The whole cell extract containing the telomerase enzyme is added to a Mastermix containing all reagents required for the reaction in a 96‐well plate including Taq polymerase, dNTPs, the upstream (TS), and downstream (ACX) primers as well as a fluorescent dye (such as SYBR green). Step 2 (Amplification) is where the extended products are amplified by Taq polymerase, utilising the upstream (TS) and downstream (ACX) primers. The ACX primer is the reverse primer and consists of a 6‐bp ‘anchor’, which caps the 3′ end of the telomerase product after the first PCR cycle to prevent further elongation of the telomerase products. Additionally, this ACX primer prevents self‐amplification, to assist in reducing primer‐dimer formation. Step 3 (Detection) of the protocol includes the detection of fluorescent signals produced by the incorporation of the fluorescent dye into the amplification products and occurs at the end of the extension step of each amplification cycle. The amount of TRAP products produced is then directly proportional to the fluorescence emission produced. (Created with BioRender.com).
**Table S1.** List of the dysregulated (both up‐and downregulated) proteins and their function in biological pathways.
**Table S2.** All downregulated proteins identified via SWATH‐MS after downregulation of LRP/LR.
**Table S3.** All upregulated proteins identified via SWATH‐MS after downregulation of LRP/LR.

## Data Availability

The data that support the findings of this study are available from the corresponding author [eloise.vandermerwe@wits.ac.za] upon reasonable request.
