# Supplementary material for: Downregulation of LRP/LR with siRNA inhibits several cancer hallmarks in lung cancer cells
Source: FEBS Open Bio. 2023 Jan 13;13(2):323–40. doi: 10.1002/2211-5463.13544 (PMC9900088; doi:10.1002/2211-5463.13544)
Supplement: Supplementary file 1 — Fig. S1. Modified Telomeric Repeat Amplification Protocol for real‐time quantification of telomerase activity. This protocol makes use of fluorescent dsDNA binding dyes which allows for detection and quantification of telomerase activity by directly measuring real‐time fluorescence emission via qPCR. Step 1 (Extension) of the reaction allows the telomerase enzyme in an extracted sample to add telomeric repeats to the 3′ end of a Telomeric Substrate (TS). The whole cell extract containing the telomerase enzyme is added to a Mastermix containing all reagents required for the reaction in a 96‐well plate including Taq polymerase, dNTPs, the upstream (TS), and downstream (ACX) primers as well as a fluorescent dye (such as SYBR green). Step 2 (Amplification) is where the extended products are amplified by Taq polymerase, utilising the upstream (TS) and downstream (ACX) primers. The ACX primer is the reverse primer and consists of a 6‐bp ‘anchor’, which caps the 3′ end of the telomerase product after the first PCR cycle to prevent further elongation of the telomerase products. Additionally, this ACX primer prevents self‐amplification, to assist in reducing primer‐dimer formation. Step 3 (Detection) of the protocol includes the detection of fluorescent signals produced by the incorporation of the fluorescent dye into the amplification products and occurs at the end of the extension step of each amplification cycle. The amount of TRAP products produced is then directly proportional to the fluorescence emission produced. (Created with BioRender.com). Table S1. List of the dysregulated (both up‐and downregulated) proteins and their function in biological pathways. Table S2. All downregulated proteins identified via SWATH‐MS after downregulation of LRP/LR. Table S3. All upregulated proteins identified via SWATH‐MS after downregulation of LRP/LR. [file FEB4-13-323-s001.docx]

**Supporting information**


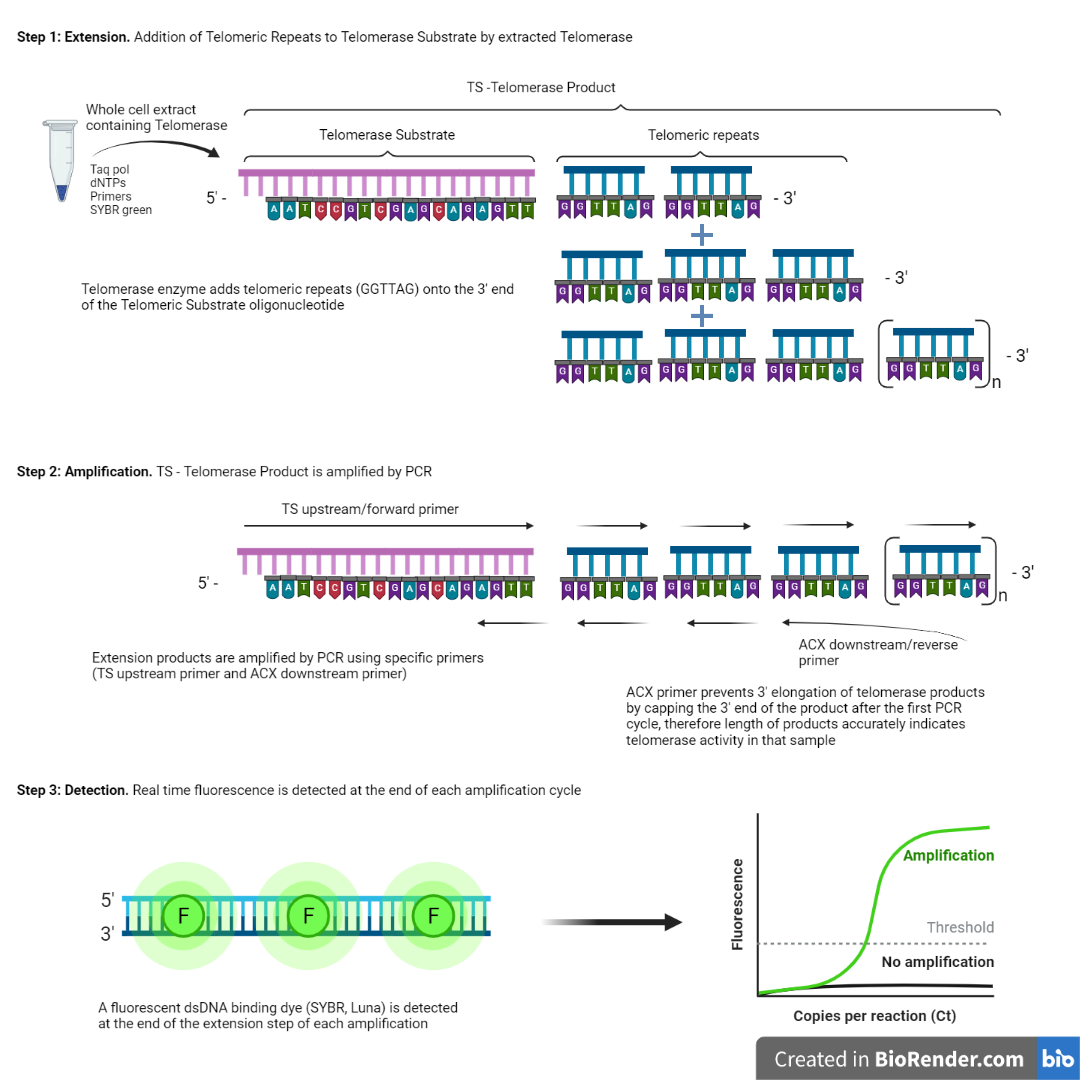


**Figure S1: Modified Telomeric Repeat Amplification Protocol for real time quantification of telomerase activity.**

*This protocol makes use of fluorescent dsDNA binding dyes which allows for detection and quantification of telomerase activity by directly measuring real time fluorescence emission via qPCR. Step 1 (****Extension****) of the reaction allows the telomerase enzyme in an extracted sample to add telomeric repeats to the 3ʹ end of a Telomeric Substrate (TS). The whole cell extract containing the telomerase enzyme is added to a Mastermix containing all reagents required for the reaction in a 96-well plate including Taq polymerase, dNTPs, the upstream (TS) and downstream (ACX) primers as well as a fluorescent dye (such as SYBR green). Step 2 (****Amplification****) is where the extended products are amplified by Taq polymerase, utilising the upstream (TS) and downstream (ACX) primers. The ACX primer is the reverse primer and consists of a 6-bp ‘anchor’, which caps the 3’ end of the telomerase product after the first PCR cycle to prevent further elongation of the telomerase products. Additionally, this ACX primer prevents self-amplification, to assist in reducing primer-dimer formation. Step 3 (****Detection****) of the protocol includes the detection of fluorescent signals produced by the incorporation of the fluorescent dye into the amplification products and occurs at the end of the extension step of each amplification cycle. The amount of TRAP products produced are then directly proportional to the fluorescence emission produced. (Created with BioRender.com).*

**Table S1: List of the dysregulated (both up-and downregulated) proteins and their function in biological pathways**

| **Fold change** | **STRING Identifier**  **(UniProt ID)** | **Protein Name** | **Pathways** |
| --- | --- | --- | --- |
| -3.7197 | ZC3H11A (O75152) | Zinc finger CCCH domain-containing protein 11A | Transcription, metabolism of RNA, meiosis |
| -1.9631 | H2AFZ (P0C0S5) | Histone H2A.Z | Cell cycle, cellular response to stress, chromatin modification, activation of HOX genes, disease of programmed cell death, base excision repair, DNA replication pre-initiation, transcription, protein metabolism, NOTCH + Wnt + RHO + nuclear receptor signal transduction |
| 1.9839 | C15orf48 (Q9C002) | Normal mucosa of esophagus-specific gene 1 protein |  |
| 2.0217 | APOL2 (Q9BQE5) | Apolipoprotein L2 | Unclarified [[81](#_ENREF_81)] |
| 2.0361 | LCN2 (P80188) | Neutrophil gelatinase-associated lipocalin | Innate immune system – cytokine signalling, iron uptake and transport |
| 2.0493 | NAPA (P54920) | Alpha-soluble NSF attachment protein | Post-translational protein modification, vesicle-mediated transport -membrane trafficking |
| 2.2511 | PNPT1 (Q8TCS8) | Polyribonucleotide nucleotidyltransferase 1, mitochondrial | Nucleotidyl group transfer [[82](#_ENREF_82)] |
| 2.3045 | PC (P11498) | Pyruvate carboxylase, mitochondrial | Disease of metabolism, metabolism of carbohydrates + vitamins and cofactors |
| 2.3345 | EIF2AK2 (P19525) | Interferon-induced, double-stranded RNA-activated protein kinase | Infectious disease, cytokine signalling |
| 2.3584 | PSMB8 (P28062) | Proteasome subunit beta type-8 | Cell cycle, cellular response to stimuli, developmental biology, disease of signal transduction and transmembrane transporters, infectious disease, DNA replication pre-initiation - DNA synthesis, adaptive and innate immune system – cytokine signalling, post-translational protein modification, regulation of mRNA stability, metabolism of amino acids, apoptosis, NOTCH signalling, Pol II transcription, ABC-family protein transport |
| 2.6578 | PSMB9 (P28065) | Proteasome subunit beta type-9 | Cell cycle, cellular response to stimuli, developmental biology, disease of signal transduction and transmembrane transporters, infectious disease, DNA replication pre-initiation – DNA synthesis, adaptive and innate immune system – cytokine signalling, post-translational protein modification, regulation of mRNA stability, metabolism of amino acids, apoptosis, NOTCH signalling, Pol II transcription, ABC-family protein transport |
| 2.7058 | PML (P29590) | Protein PML | Infectious disease, cytokine signalling, Post- translational protein modification, Pol II transcription |
| 2.7725 | TAP1 (Q03518) | Antigen peptide transporter 1 | Adaptive immune system |
| 2.9067 | LGALS3BP (Q08380) | Galectin-3-binding protein | Haemostasis -platelet activation, signalling and aggregation |
| 2.9820 | IFIT2 (P09913) | Interferon-induced protein with tetratricopeptide repeats 2 | Cytokine signalling |
| 3.0249 | TYMP (P19971) | Thymidine phosphorylase | Metabolism of nucleotides |
| 3.0590 | ISG20 (Q96AZ6) | Interferon-stimulated gene 20 kDa protein | Cytokine signalling |
| 3.1678 | NT5C3A (Q9H0P0) | Cytosolic 5'-nucleotidase 3A | Metabolism of nucleotides |
| 3.1814 | LAP3 (P28838) | Cytosol aminopeptidase | Lysosomal proteolytic degradation [[83](#_ENREF_83)] |
| 3.1882 | TAPBP (O15533) | Tapasin | Adaptive immune system - |
| 3.3612 | SAMHD1 (Q9Y3Z3) | Deoxynucleoside triphosphate triphosphohydrolase | Cytokine signalling, metabolism of nucleotides |
| 3.4444 | IFI35 (P80217) | Interferon-induced 35 kDa protein | Cytokine signalling |
| 3.4587 | OAS3 (Q9Y6K5) | 2'-5'-oligoadenylate synthase 3 | Cytokine signalling |
| 3.5885 | STAT1 (P42224) | Signal transducer and activator of transcription 1 | Disease of signal transduction, cytokine signalling, RTK and NOTCH signalling, Pol II transcription |
| 3.9890 | HLA-A (P04439) | HLA class I histocompatibility antigen, A-3 alpha chain | Adaptive immune system – cytokine signalling, post-translational protein modification |
| 4.9251 | HLA-B (P01889) | HLA class I histocompatibility antigen, B-7 alpha chain | Adaptive and innate immune system – cytokine signalling |
| 5.2837 | DDX58 (O95786) | Probable ATP-dependent RNA helicase | Innate immune system – cytokine signalling, post-translational protein modification |
| 5.6279 | OAS1 (P00973) | 2'-5'-oligoadenylate synthase 1 | Cytokine signalling |
| 5.6556 | IFIT3 (O14879) | Interferon-induced protein with tetratricopeptide repeats 3 | Cytokine signalling |
| 6.5920 | OASL (Q15646) | 2'-5'-oligoadenylate synthase-like protein | Cytokine signalling |
| 6.7055 | ISG15 (P05161) | Ubiquitin-like protein ISG15 | Infectious disease, DNA repair- DNA damage bypass, innate immune system – cytokine signalling |
| 7.2337 | IFIT1 (P09914) | Interferon-induced protein with tetratricopeptide repeats 1 | Cytokine signalling |

^a^ Pathway/function of the proteins presented were identified using publicly available databases, such as UniProt ([http://www.uniprot.org](http://www.uniprot.org/)), STRING database (<https://string-db.org>) and Reactome pathway browser (<https://reactome.org/PathwayBrowser/#TOOL=AT>).

**Table S2: All downregulated proteins identified via SWATH-MS after downregulation of LRP/LR**

| **Fold change** | **Protein Identifier**  **(UniProt ID)** | **Protein Name** | **p-value** | **Function** |
| --- | --- | --- | --- | --- |
| **^a^-3.7197** | **ZC3H11A (O75152)** | **Zinc finger CCCH domain-containing protein 11A** | **0.012118** | **Involved in nuclear mRNA export** |
| **-1.9631** | **H2AFZ (P0C0S5)** | **Histone H2A.Z** | **0.000631** | **Variant histone H2A which replaces conventional H2A in a subset of nucleosomes** |
| -1.4441 | HPGD (P15428) | 15-hydroxyprostaglandin dehydrogenase [NAD (+)] | 7.59E-09 | -Prostaglandin inactivation  -Catalyses the NAD-dependent dehydrogenation of lipoxin A4 to form 15-oxo-lipoxin A4  -Inhibits in vivo proliferation of colon cancer cells |
| -1.3834 | LAMP1 (P11279) | Lysosome-associated membrane glycoprotein 1 | 1.24E-06 | -Presents carbohydrate ligands to selectins.  -Implicated in tumour cell metastasis |
| -1.3683 | ACO1 (P21399) | Cytoplasmic aconitate hydratase | 6.62E-10 | -Binds a 4Fe-4S cluster and functions as aconitase when cellular iron levels are high  -Functions as mRNA binding protein that regulates uptake, sequestration and utilization of iron when cellular iron levels are low  -Binds to iron-responsive elements (IRES) in target mRNA species when iron levels are low |
| -1.3569 | LAMTOR1 (Q6IAA8) | Ragulator complex protein LAMTOR1 | 0.000213 | Involved in amino acid sensing and activation of mTORC1, a signalling complex promoting cell growth in response to growth factors, energy levels, and amino acids |
| -1.3474 | CTSD (P07339) | Cathepsin D | 1.10E-08 | -Acid protease active in intracellular protein breakdown  -Involved in the pathogenesis of several diseases such as breast cancer and possibly Alzheimer disease |
| -1.3157 | HRSP12 (P52758) | 2-iminobutanoate/2-iminopropanoate deaminase | 0.003533 | Catalyzes the hydrolytic deamination of enamine/imine intermediates that form during normal metabolism |
| -1.2833 | GBE1 (Q04446) | 1,4-alpha-glucan-branching enzyme | 4.02E-07 | Required for normal glycogen accumulation |
| -1.2697 | GCLC (P48506) | Glutamate-cysteine ligase catalytic subunit | 5.41E-08 |  |
| -1.2633 | GBA (P04062) | Glucosylceramidase | 0.000575 | -Plays a central role in the degradation of complex lipids and the turnover of cellular membranes  -Plays a role in cholesterol metabolism |
| -1.2419 | CNTN1 (Q12860) | Contactin-1 | 4.02E-07 | -Mediate cell surface interactions during nervous system development |
| -1.2083 | TIMM9 (Q9Y5J7) | Mitochondrial import inner membrane translocase subunit | 0.000288 | Mitochondrial intermembrane chaperone that participates in the import and insertion of multi-pass transmembrane proteins into the mitochondrial inner membrane |
| -1.1543 | PCYOX1 (Q9UHG3) | Prenylcysteine oxidase 1 | 0.000215 | -Involved in the degradation of prenylated proteins. |
| -1.1333 | DDX27 (Q96GQ7) | Probable ATP-dependent RNA helicase | 4.62E-06 | -Component of the nucleolar ribosomal RNA (rRNA) processing machinery that regulates 3' end formation of ribosomal 47S rRNA |
| -1.0995 | UBE3C (Q15386) | Ubiquitin-protein ligase E3C | 1.09E-05 | E3 ubiquitin-protein ligase that accepts ubiquitin from the E2 ubiquitin-conjugating enzyme UBE2D1 in the form of a thioester and then directly transfers the ubiquitin to targeted substrates |
| -1.0725 | HNRNPUL1 (Q9BUJ2) | Heterogeneous nuclear ribonucleoprotein U-like protein 1 | 2.07E-06 | -Acts as a basic transcriptional regulator. Represses basic transcription driven by several virus and cellular promoters. When associated with BRD7, activates transcription of glucocorticoid-responsive promoter in the absence of ligand- stimulation.  -Plays a role in mRNA processing and transport |
| -1.0429 | PARP14 (Q460N5) | Poly [ADP-ribose] polymerase 14 | 0.001432 | -By mono-ADP-ribosylating STAT1 at 'Glu-657' and 'Glu-705' and thus decreasing STAT1 phosphorylation  -Negatively regulates pro-inflammatory cytokines production in macrophages in response to IFNG stimulation. |
| -1.0416 | PRKAR2A (P13861) | cAMP-dependent protein kinase type II-alpha regulatory subunit | 0.000303 | -involved in cAMP signalling in cells  -Type II regulatory chains mediate membrane association by binding to anchoring proteins, including the MAP2 kinase |
| -1.0155 | RAD23B (P54727) | UV excision repair protein RAD23 homolog B | 2.64E-06 | Multiubiquitin chain receptor involved in modulation of proteasomal degradation |
| -1.0023 | DBI (P07108) | Acyl-CoA-binding protein | 1.93E-06 | Binds medium- and long-chain acyl-CoA esters with very high affinity and may function as an intracellular carrier of acyl-CoA esters |

^a^ proteins in **bold** are those with a fold change of 2 and above.

**Table S3: All upregulated proteins identified via SWATH-MS after downregulation of LRP/LR**

| **Fold change** | **STRING Identifier**  **(UniProt ID)** | **Protein Name** | **p-value** | **Function** |
| --- | --- | --- | --- | --- |
| 1.0380 | NDUFB10  (O96000) | NADH dehydrogenase [ubiquinone] 1 beta subcomplex subunit 10 | 4.20E-09 | Accessory subunit of the mitochondrial membrane respiratory chain NADH dehydrogenase (Complex I) |
| 1.0593 | NUB1 (Q9Y5A7) | NEDD8 ultimate buster 1 | 3.25E-05 | Specific down-regulator of the NEDD8 conjugation system |
| 1.1022 | KPNA2 (P52292) | Importin subunit alpha-1 | 6.89E-06 | Functions in nuclear protein import as an adapter protein for nuclear receptor KPNB1 |
| 1.1092 | NCOR2 (Q9Y618) | Nuclear receptor corepressor 2 | 0.00343 | Mediates the transcriptional repression activity of some nuclear receptors by promoting chromatin condensation |
| 1.1113 | RRBP1 (Q9P2E9) | Ribosome-binding protein 1 | 1.19E-06 | Acts as a ribosome receptor and mediates interaction between the ribosome and the endoplasmic reticulum membrane |
| 1.1190 | CYCS (P99999) | Cytochrome c | 3.15E-09 | Electron carrier protein |
| 1.1424 | SQRDL (Q9Y6N5) | Sulfide:quinone oxidoreductase, mitochondrial | 1.47E-08 | Catalyses the oxidation of hydrogen sulphide with the help of a quinone, such as ubiquinone, giving rise to thiosulfate and ultimately to sulfane (molecular sulphur) atoms. |
| 1.1589 | RNF213 (Q63HN8) | E3 ubiquitin-protein ligase | 1.05E-05 | Involved in angiogenesis -Involved in the non-canonical Wnt signalling pathway in vascular development |
| 1.1594 | MIA2 (Q96PC5) | Melanoma inhibitory activity 2 | 0.000265 |  |
| 1.1757 | TST (Q16762) | Thiosulfate sulfotransferase | 0.000148 | Formation of iron-sulfur complexes, cyanide detoxification or modification of sulfur-containing enzymes |
| 1.1872 | ZC3HAV1 (Q7Z2W4) | Zinc finger CCCH-type antiviral protein 1 | 2.50E-07 | Antiviral protein which inhibits the replication of viruses by recruiting the cellular RNA degradation machineries to degrade the viral mRNAs |
| 1.2062 | MRPS36 (P82909) | Mitochondrial ribosomal protein S36 | 0.014676 |  |
| 1.3110 | EPHA2 (P29317) | Ephrin type-A receptor 2 | 0.007786 | Receptor tyrosine kinase which binds promiscuously membrane-bound ephrin-A family ligands residing on adjacent cells |
| 1.3332 | LIMA1 (Q9UHB6) | LIM domain and actin-binding protein 1 | 7.65E-06 | Binds to actin monomers and filaments. ---Increases the number and size of actin stress fibres and inhibits membrane ruffling  - Inhibits actin filament depolymerization. Bundles actin filaments, delays filament nucleation and reduces formation of branched filaments |
| 1.3612 | PSME2 (Q9UL46) | Proteasome activator complex subunit 2 | 2.43E-07 | Implicated in immunoproteasome assembly and required for efficient antigen processing |
| 1.5087 | PSME1 (Q06323) | Proteasome activator complex subunit 1 | 1.82E-11 | Implicated in immunoproteasome assembly and required for efficient antigen processing. |
| 1.5478 | SPATS2L (Q9NUQ6) | Spermatogenesis associated serine rich 2 like protein | 5.12E-09 | Belongs to the SPATS2 family |
| 1.5699 | EHD4 (Q9H223) | EH domain-containing protein 4 | 4.80E-07 | ATP- and membrane-binding protein that probably controls membrane reorganization/tubulation upon ATP hydrolysis |
| 1.6533 | MRPL15 (Q9P015) | Mitochondrial ribosomal protein L15 | 2.06E-06 | is a member of MRPL, which provides energy in the form of ATP for cell growth |
| 1.6601 | STK4 (Q13043) | Serine/threonine-protein kinase 4 | 9.50E-05 | -Stress-activated, pro-apoptotic kinase which, following caspase-cleavage, enters the nucleus and induces chromatin condensation followed by internucleosomal DNA fragmentation  - Key component of the Hippo signalling pathway which plays a pivotal role in organ size control and tumour suppression by restricting proliferation and promoting apoptosis |
| 1.7610 | TGM2 (P21980) | Protein-glutamine gamma-glutamyltransferase 2 | 0.000436 | Catalyses the cross-linking of proteins and the conjugation of polyamines to proteins |
| 1.7854 | CNP (P09543) | 2',3'-cyclic-nucleotide 3'-phosphodiesterase | 2.42E-07 | May participate in RNA metabolism in the myelinating cell |
| 1.8448 | OGFR (Q9NZT2) | Opioid growth factor receptor | 1.35E-09 | Seems to be involved in growth regulation |
| **^a^1.9839** | **C15orf48 (Q9C002)** | **Normal mucosa of esophagus-specific gene 1 protein** | **1.34E-07** | **Belongs to the complex I NDUFA4 subunit family** |
| **2.0217** | **APOL2 (Q9BQE5)** | **Apolipoprotein L2** | **1.19E-06** | **May affect the movement of lipids in the cytoplasm or allow the binding of lipids to organelles** |
| **2.0361** | **LCN2 (P80188)** | **Neutrophil gelatinase-associated lipocalin** | **0.015405** | **Iron-trafficking protein involved in multiple processes such as apoptosis, innate immunity and renal development** |
| **2.0493** | **NAPA (P54920)** | **Alpha-soluble NSF attachment protein** | **2.43E-08** | **Required for vesicular transport between the endoplasmic reticulum and the Golgi apparatus** |
| **2.2511** | **PNPT1 (Q8TCS8)** | **Polyribonucleotide nucleotidyltransferase 1, mitochondrial** | **3.02E-08** | **RNA-binding protein implicated in numerous RNA metabolic processes** |
| **2.3045** | **PC (P11498)** | **Pyruvate carboxylase, mitochondrial** | **0.002444** | **Catalyses in a tissue specific manner, the initial reactions of glucose (liver, kidney) and lipid (adipose tissue, liver, brain) synthesis from pyruvate** |
| **2.3345** | **EIF2AK2 (P19525)** | **Interferon-induced, double-stranded RNA-activated protein kinase** | **4.52E-08** | **Plays a key role in the innate immune response to viral infection and is also involved in the regulation of signal transduction, apoptosis, cell proliferation and differentiation** |
| **2.3584** | **PSMB8 (P28062)** | **Proteasome subunit beta type-8** | **1.28E-05** | **Involved in antigen processing to generate class I binding peptides** |
| **2.6578** | **PSMB9 (P28065)** | **Proteasome subunit beta type-9** | **5.99E-10** | **Involved in antigen processing to generate class I binding peptides** |
| **2.7058** | **PML (P29590)** | **Protein PML** | **0.000747** | **Functions via its association with PML-nuclear bodies (PML-NBs) in a wide range of important cellular processes, including tumour suppression, transcriptional regulation, apoptosis, senescence, DNA damage response, and viral defence mechanisms** |
| **2.7725** | **TAP1 (Q03518)** | **Antigen peptide transporter 1** | **4.03E-08** | **Involved in the transport of antigens from the cytoplasm to the endoplasmic reticulum for association with MHC class I molecules** |
| **2.9067** | **LGALS3BP (Q08380)** | **Galectin-3-binding protein** | **4.03E-07** | **- Promotes integrin-mediated cell adhesion**  **- May stimulate host defence against viruses and tumour cells** |
| **2.9820** | **IFIT2 (P09913)** | **Interferon-induced protein with tetratricopeptide repeats 2** | **0.008082** | **- inhibits expression of viral messenger RNAs lacking 2'-O-methylation of the 5' cap**  **- provide a molecular signature to distinguish between self and non-self mRNAs by the host during viral infection** |
| **3.0249** | **TYMP (P19971)** | **Thymidine phosphorylase** | **4.54E-10** | **- May have a role in maintaining the integrity of the blood vessels**  **- Has growth promoting activity on endothelial cells, angiogenic activity in vivo and chemotactic activity on endothelial cells in vitro** |
| **3.0590** | **ISG20 (Q96AZ6)** | **Interferon-stimulated gene 20 kDa protein** | **3.51E-06** | **Interferon-induced antiviral exoribonuclease that acts on single-stranded RNA and also has minor activity towards single- stranded DNA** |
| **3.1678** | **NT5C3A (Q9H0P0)** | **Cytosolic 5'-nucleotidase 3A** | **1.31E-10** | **Nucleosidase which shows specific activity towards cytidine monophosphate (CMP) and 7-methylguanosine monophosphate (m(7)GMP)** |
| **3.1814** | **LAP3 (P28838)** | **Cytosol aminopeptidase** | **2.52E-08** | **- Presumably involved in the processing and regular turnover of intracellular proteins**  **- Catalyses the removal of unsubstituted N-terminal amino acids from various peptides** |
| **3.1882** | **TAPBP (O15533)** | **Tapasin** | **2.37E-10** | **Involved in the association of MHC class I with transporter associated with antigen processing (TAP) and in the assembly of MHC class I with peptide (peptide loading)** |
| **3.3612** | **SAMHD1 (Q9Y3Z3)** | **Deoxynucleoside triphosphate triphosphohydrolase** | **6.29E-08** | **Host restriction nuclease involved in defence response to virus** |
| **3.4444** | **IFI35 (P80217)** | **Interferon-induced**  **35 kDa protein** | **2.65E-10** | **Not yet known** |
| **3.4587** | **OAS3 (Q9Y6K5)** | **2'-5'-oligoadenylate synthase 3** | **5.64E-06** | **-Interferon-induced, dsRNA-activated antiviral enzyme which plays a critical role in cellular innate antiviral response**  **- may also play a role in other cellular processes such as apoptosis, cell growth, differentiation and gene regulation** |
| **3.5885** | **STAT1 (P42224)** | **Signal transducer and activator of transcription 1** | **1.88E-09** | **Signal transducer and transcription activator that mediates cellular responses to interferon-alpha, interferon-gamma, EGF, PDGF and IL6**  **-mediates the expression of a variety of genes, which is thought to be important for cell viability in response to different cell stimuli and pathogens** |
| **3.9890** | **HLA-A (P04439)** | **HLA class I histocompatibility antigen, A-3 alpha chain** | **2.02E-09** | **Involved in the presentation of foreign antigens to the immune system** |
| **4.9251** | **HLA-B (P01889)** | **HLA class I histocompatibility antigen, B-7 alpha chain** | **4.93E-07** | **Involved in the presentation of foreign antigens to the immune system** |
| **5.2837** | **DDX58 (O95786)** | **Probable ATP-dependent RNA helicase** | **7.02E-09** | **Innate immune receptor which acts as a cytoplasmic sensor of viral nucleic acids and plays a major role in sensing viral infection and in the activation of a cascade of antiviral responses including the induction of type I interferons and proinflammatory cytokines** |
| **5.6279** | **OAS1 (P00973)** | **2'-5'-oligoadenylate synthase 1** | **1.80E-10** | **Interferon-induced, dsRNA-activated antiviral enzyme which plays a critical role in cellular innate antiviral response**  **- may also play a role in other cellular processes such as apoptosis, cell growth, differentiation and gene regulation** |
| **5.6556** | **IFIT3 (O14879)** | **Interferon-induced protein with tetratricopeptide repeats 3** | **1.52E-12** | **FN-induced antiviral protein which acts as an inhibitor of cellular as well as viral processes, cell migration, proliferation, signalling, and viral replication.** |
| **6.5920** | **OASL (Q15646)** | **2'-5'-oligoadenylate synthase-like protein** | **5.85E-08** | **Does not have 2'-5'-OAS activity, but can bind double- stranded RNA**  **- antiviral activity**  **-paralog of OAS1** |
| **6.7055** | **ISG15 (P05161)** | **Ubiquitin-like protein ISG15** | **8.41E-11** | **Ubiquitin-like protein which plays a key role in the innate immune response to viral infection either via its conjugation to a target protein (ISGylation) or via its action as a free or unconjugated protein** |
| **7.2337** | **IFIT1 (P09914)** | **Interferon-induced protein with tetratricopeptide repeats 1** | **5.21E-12** | **Interferon-induced antiviral RNA-binding protein that specifically binds single-stranded RNA bearing a 5'-triphosphate group (PPP-RNA), thereby acting as a sensor of viral single- stranded RNAs and inhibiting expression of viral messenger RNAs** |

^a^ proteins in **bold** are those with a fold change of 2 and above.
